# Acoustic and vibrational signaling in true katydid *Nesoecia nigrispina*: three means of sound production in one species

**DOI:** 10.7717/peerj.13749

**Published:** 2022-07-14

**Authors:** Olga S. Korsunovskaya, Rustem D. Zhantiev

**Affiliations:** Department of Entomology, Lomonosov Moscow State University, Moscow, Russia

**Keywords:** Orthoptera, Tettigoniidae, Pseudophyllinae, *Nesoecia nigrispina*, Acoustic signals, Vibratory signals, Tremulation

## Abstract

The males of Mexican katydids *Nesoecia nigrispina* (Stal, 1873) produce calling songs and protest sounds using the typical stridulatory apparatus, situated, as in most of the other Ensifera, at the bases of the tegmina. It includes a stridulatory file on the upper tegmen and a plectrum on the lower one. The calling sounds, which are of two types (fast and slow), are two-syllabic series, with a repetition rate fluctuate within 3–4.5 s^−1^ (fast) and 1.2–2 s^−1^ (slow). After tactile stimulation, males produce protest signals in the form of short trills of uniform syllable duration. The syllable repetition rate is higher than that of the calling sounds: 7.7 s^−1^. The frequency spectra of these signals have maxima in the band of 14–15 kHz. However, in addition to the sounds described, both males and females are capable of producing protest signals of the second type, with the help of another sound apparatus, namely the hind wings. Apparently, the sound is produced by the friction of the hind wings on the lower tegmen. The dominant frequencies in the frequency spectra of these sounds are 40–60 kHz. In adults of both sexes and older nymphs, in response mainly to tactile stimulation, short clicks are recorded, which they produce, apparently, by the mandibles. Thus, *N. nigrispina* seems to have the most extensive acoustic repertoire among pseudophyllines and three means of emitting sound signals. Tremulatory substrate-borne vibrations are produced by individuals of both sexes during courtship and by males completing the calling signal cycle and after copulation. It is possible that vibrational signals are an additional factor in the reproductive isolation of sympatric species, since the calling sound signals in representatives of the genus *Nesoecia* are similar and exhibit considerable variability. The type and parameters of the calling signal used by the female during recognizing a conspecific mate remain unclear.

## Introduction

Katydids of the large subfamily Pseudophyllinae (true katydids) are common in the tropics and subtropics in both the Old and New Worlds. Many of them mimic the leaves of plants, but a large group of these insects has a cryptic coloration and do not look like plants. The latter include representatives of the genus *Nesoecia*. They are large, earthy colored katydids with abbreviated wings. Currently, eigth species with minor morphological differences from southern Mexico, Brazil, and the Galapagos Islands (Floreana, one species) have been described (Cigliano et al., 2022). Relatively little is known about the biology of true katydids. Acoustic signals (airborne sounds) have been studied in some species of the tribes Cocconotini, Pleminiini and some others, in some cases they are supplemented or accompanied by substrate-borne vibrations from tremulation or substrate drumming (Belwood & Morris, 1987; Belwood, 1990; Morris et al., 1994; Roemer, Lang & Hartbauer, 2010; Rajaraman et al., 2015). Some species possess unique sound apparatus, *i.e.,* the females of *Panoploscelis specularis* (Montealegre-Z, Guerra & Morris, 2009) or sound emission mechanisms unusual for katydids, *i.e.,* the abdomino-alary in *Pantecphylus cerambycinus* (Heller, 1996). In this study we used specimens of *Nesoecia nigrispina* from a laboratory culture. This allowed us to study in detail their behavior and signaling the insects use both sound and vibratory signals, and to reveal three means of sound production in this species that they use in different behavioral contexts.

## Materials & Methods

We used *Nesoecia nigrispina* (Stal, 1873) (Orthoptera, Pseudophyllinae, Cocconotini) from the laboratory culture of the Moscow Zoo and the Department of Entomology of Lomonosov Moscow State University. The basis of the culture was a female katydid caught in Mexico, in the northern half of the Yucatan Peninsula (Chechen Itza), under a tree bark then and presented to the insectarium of the Moscow Zoo. The lifetime of one generation is about a year, but in nature its duration is apparently shorter, because in the last 1–1.5 months of life, the insects become inactive, sedentary and often lose one or two legs, obviously, it makes them an easy prey for predators. A female (adult phase), lives for at least 6 months, mates and lays eggs during almost all her life. The development of the nymphs lasts about four months at 25 °C. By now, 5–6 generations have already passed—the descendants of the first female individual. Katydids were kept in insect cages 30 × 30 × 30 cm. Both adults and nymphs of different age can be kept together in one box. Cannibalism is not typical for this species. The fodder was raspberry, blackberry, oak, and lettuce leaves, in summer—clover and dandelion leaves, fruit slices (orange, apple), oatmeal and protein supplements in a form of dried freshwater amphipods of the genus *Gammarus*. For egg-laying, females were offered containers with a wet mixture of peat and soil. *N. nigrispina* are nocturnal insects, therefore we did not register singing during daytime. At daylight hours, insects sat on the walls of their cages or pieces of wood, as a rule, completely motionless. The katydids were reared at temperature of 25–27 °C and under a constant photoperiod 12 L:12 D. Sound signals of *N. nigrispina* were recorded digitally using a Roland R-05 Digital Audio Recorder (frequency response 0.02–40 kHz, flat response 0.02–20 kHz) with a sampling rate of 96 kHz or using 1/4″ Brüel & Kjær microphone 4135 (flat response 0.01–70 kHz) and Bruel and Kjaer low-noise measuring amplifier 2606 (frequency range 0.002–200 kHz ± 0,5 dB re 1 kHz) with a sampling rate of 200 kHz. Amplifier was connected *via* an analogue-to-digital converter E-14-440 (L-Card, Russia) to a PC. In some cases, a Magenta bat detector Mk5 V 2.0 44 (0) 1283 565435 (England) was used to monitor katydid’s acoustic activity. Vibratory signals of *N. nigrispina* were recorded using a ZPK-56 pickup head (Russia) with a piezoceramic element made of barium titanate (frequency response 30–12,000 Hz) mounted horizontally with the needle stand upside down. This device was connected *via* the same analogue-to-digital converter to PC. Digitizing frequency at recordings of vibrational signals was 5 kHz. The signals were recorded using the program PowerGraph 3.3. (DiSoft, Russia). Acoustic and vibratory signals were processed using the CoolEditPro 2.1 and PowerGraph 3.3 softwares. For Fast Fourier Transformation, the Power Graph program was used with FFT size of 16384 lines. If necessary, a band stop filter was used to eliminate 50 Hz interference.

Night recordings were performed using a Nikon 1J4 video camera without an infrared filter under the light of an Orient SAL-130C infrared illuminator. High-speed video filming was carried out with a frame rate of 1,000 per second.

Scanning electron micrographs (SEMs) of specimens were made with a JEOL/EO scanning electron microscope (Japan) (Electron Microscopy Laboratory, Faculty of Biology, Lomonosov Moscow State University). Photographs and measurements of morphological characters were made with a using a Canon EOS 6D digital camera with a Canon MP-E65 macro lens.

Sound and vibrational signal recordings were performed at 25–26 °C. The limits of temporal parameters of the sound signals are given according to the data on the signals of 5–7 insects, the mean values and standard deviations are given according to the data of the signals of one insect, so as not to overestimate the variability of temporal parameters of the signal of a particular insect.

Means and standard deviations were calculated using ≥20 measurements. A total of 49 records of 25 males and 12 females were analyzed.

### Terminology

To describe sound and vibratory signals we mainly follow bioacoustic terminology used by Heller et al. (2021): calling song—spontaneous song produced by an isolated male. Syllable—the sound produced by one complete up (opening) and down (closing) stroke of the forewings (tegmina) or hind wings. Tooth-impact—short sound impulse arising during the contact of a single tooth of a stridulatory file (pars stridens) with a plectrum. Click—fast train of sound waves, which arise at strucking any structures. Series—group of several syllable. The main elements of the temporal pattern of signals are further marked in the corresponding figures.

## Results

Proceeding from the fact that Mexican and South American species of *Nesoecia* morphologically are very similar, we considered necessary to describe some characters of male and female katydids, which we identified as *N. nigrispina*.

*N. insignis* Hebard, 1932, described a male individual, morphologically is the closest species to *N. nigrispina*. They differ in body size, stridulatory area of a male’s left tegmen, proportions of a male’s subgenital plate, and ovipositor shape (Hebard, 1932; Cigliano et al., 2022). The katydids we have studied are larger than the type specimen of *N. nigrispina*, however, some features match the morphological characters of *N. nigrispina*: venation of tegmina, smaller size of male’s tegminal stridulatory area, shape of a pronotum, straight and shorter ovipositor, than in *N. insignis* females. The objects of our study differ from other Mexican species in acoustic signals.

**Figure 1:**
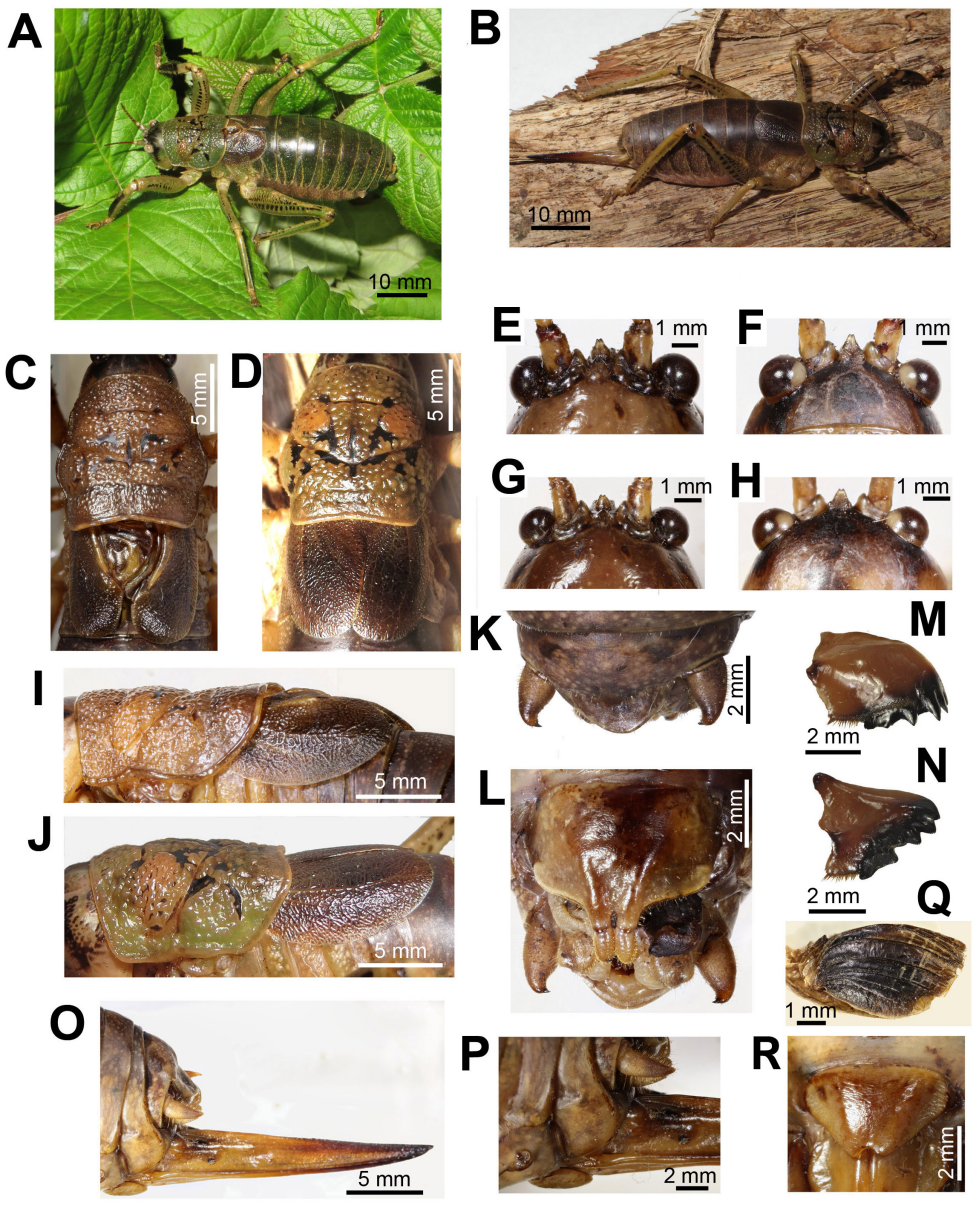
Habitus and body details of the male and female of *Nesoecia nigrispina*. (A) Male habitus. (B) Female habitus. (C) Male pronotum and tegmina, dorsal view. (D) Female pronotum and tegmina, dorsal view. (E) Male vertex and fastigium, anterior view. (F) The same, dorsal view. (G) Female vertex and fastigium, anterior view. (H) The same, dorsal view; (I) Male pronotum and tegmina, lateral view. (J) Female pronotum and tegmina, lateral view. (K) Male anal plate and cerci. (L) Male subgenital plate with stili. (M) Female left mandible, outside view. (N) Female right mandible, inside view. (O) Ovipositor. (P) Female’s terminalia: cerci and subgenital plate, lateral view. (Q) Left male hind wing, ventral view. (R) Female subgenital plate.

**Male** (Fig. 1A). Body color brown, sometimes olive brown. Head round, fastigium triangular with sinuate apex, frons with two dark spots in the upper third (Figs. 1E and 1F).

Pronotum coarsely granulated with black spots and short interrupted bands (Fig. 1C). Lateral lobes of pronotum wider than high (Fig. 1I). Lower margin of lateral lobes with small notch before the middle (Fig. 1I). Tegmina dark brown. Cell below the stridulatory file yellowish (Figs. 1C and 1I). Wing apices reach the end of the 2nd abdominal tergite. Legs on lower surface with spines. Lateral surfaces of femora and partly of tibiae with lines of dark spots. Anterior surface of fore tibia dark brown or black (Fig. 1A). Anal plate wide-triangular, cerci at apex curved inward, their length approximately 1.5 times exceeds their maximum width, bear one short apical spine (Fig. 1K). Subgenital plate is transverse; its width is 1.5 times its height; bifurcated distal part is stretched out and bears styli (Fig. 1L).

Length, mm: body 41–47, pronotum 11–12, hind femora 15–17 (*n* = 5).

**Female** (Fig. 1B). Coloration and granulation like in males. Head, and frons like in males (Figs. 1G and 1H). Pronotum is more olive in comparison to males (Figs. 1D and 1J). Lower margin of lateral lobes without notch before the middle (Fig. 1D). Tegmina unicolored, brown, do not reach the end of abdominal tergite 2. Leg coloration like in males. Ovipositor straight (Fig. 1O). Its length is approximately equal to that of the hind femur. Subgenital plate wide triangular, apically with small notch (Fig. 1R).

Length, mm: body 47–52, pronotum 11.5–13.0, hind femora (lower edge) 19–22, ovipositor (from subgenital plate to the tip) 18–20 (*n* = 5).

### Acoustic signals

*N. nigrispina* katydids emit sound signals in three ways: using a tegminal sound apparatus (males only), hind wings, and, presumably, mandibles (individuals of both sexes and nymphs).

**Figure 2:**
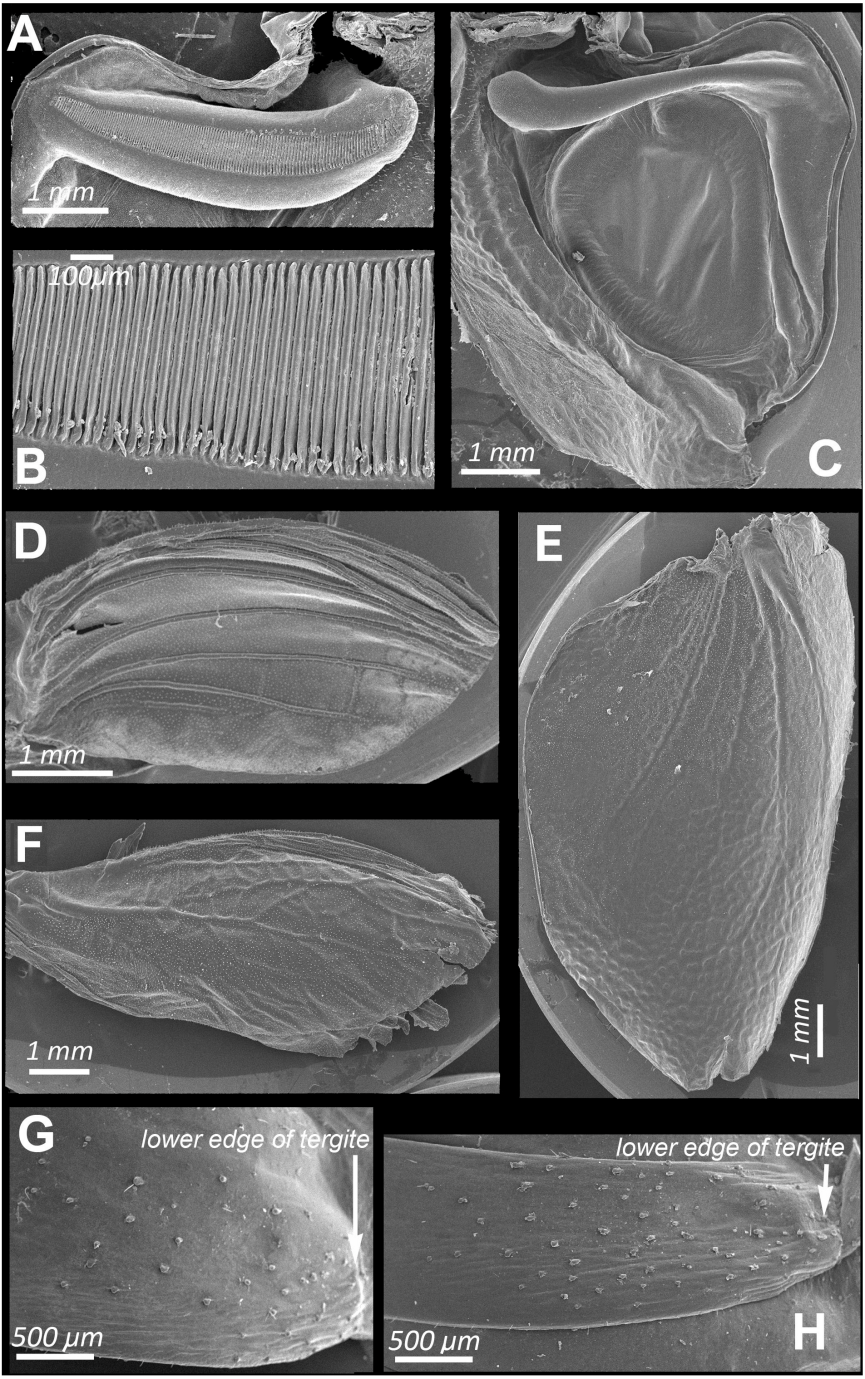
SEM images of the structures providing sound emission in *N. nigrispina*. (A, B) Male stridulatory file. (C) Male right tegmen, ventral view. (D) Right male hind wing, dorsal view. (E) Right female tegmen, ventral view; (F) Right female hind wing, dorsal view. (G) Spinules on lateral part of the 2nd male abdominal tergite. (H) The same of the 2nd female abdominal tergite.

Male calling songs are represented by long sequences of series, which therefore consist of two syllables. Insects produce them with the help of a stridulation apparatus, a typical structure for the tegminal sound apparatus of katydids. On the left tegmen, a stridulatory file (pars stridens) (Figs. 2A and 2B) is located, bearing 127–140 (*N* = 5) teeth that rub against a plectrum at anal margin of lower elytron (Fig. 2C). A resonating structure is located on right tegmen—a mirror, while the vein, which plays the role of pars stridens on the left tegmen, is thickened, but does not bear teeth (Fig. 2C).

**Figure 3:**
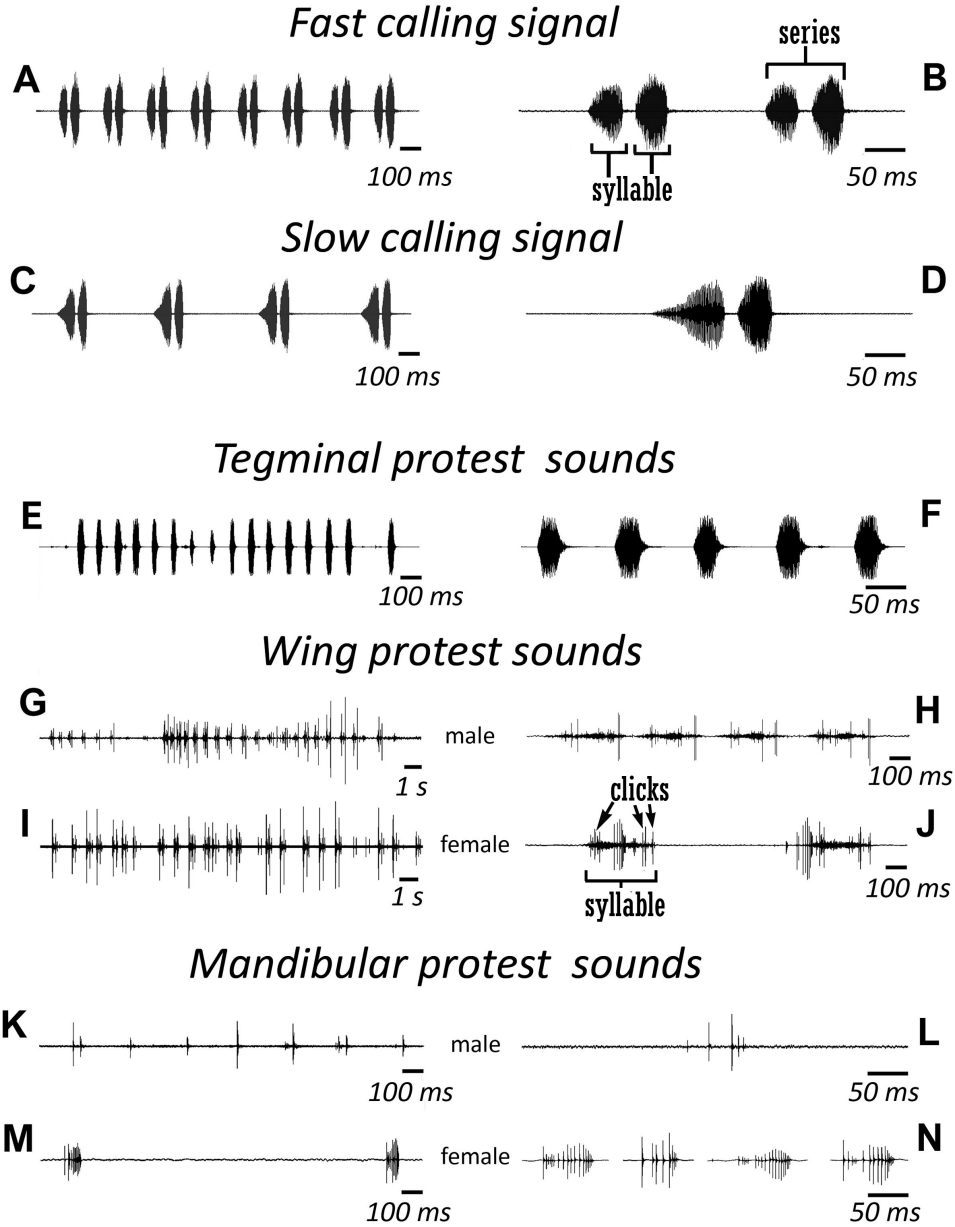
Oscillograms of the sound signals of *N. nigrispina*. (A, B) Fast calling signal. (C, D) Slow calling signal. (E, F) Tegminal protest sounds. (G, H) Male wing protest sounds. (I, J) Female wing protest sounds. (K, L) Male mandibular protest sounds. (M, N) Female mandibular protest sounds. Right panel (B, D, F, H, J, L, N)—oscillograms at higher speed.

Male’s calling can continue for several hours. They are capable to emit a calling song with two rhythms: fast (Figs. 3A and 3B; Signal S1) and slow (Figs. 3C and 3D; Signal S2), yet the insects at the beginning of a signal with a greater duty cycle, most commonly produce several series or several tens of series with a fast rhythm (Signal S2). At simultaneous singing of several katydids, their acoustic interaction was observed, it was expressed in (i) acceleration of the rhythm of signal emitting, (ii) alternation, (iii) synchronization, (iv) emitting signals identical in amplitude-temporal pattern with tegminal protest sounds. As has been shown by a high-speed video filming, males produce a series of calling signals with two cycles of opening–closing of their tegmina, while after the initial tegminal opening, a complete closure does not occur at the end of the first syllable. It was observed only after the second syllable. Form and duration of a syllable in the series, following in a fast and slow rhythm, are different. In fast rhythm series, rise and fall times of an amplitude of the first and second syllables are approximately the same, duration of the first syllable is 44.7 (SD = 2.1) ms, the second syllable—43.4 (SD = 3.2) ms. Repetition rate of the series can fluctuate within 3–4.5 s^−1^, on average it is 4.2 (SD = 0.2) s^−1^. Syllable repetition rate in the series averages 16.5 (SD = 0.3) s^−1^. For slow calling signal, duration of the first syllable is 96.5 (SD = 4.1) ms, the second syllable—47.2 (SD = 3.9) ms, rise time of the amplitude of the first syllable is much longer than the time of its fall and takes about 2/3 of syllable duration. Shape of the second syllable is approximately the same as in a series with a fast rhythm. Repetition rate of the series can vary within 1.2–2 s^−1^, mean is 1.86 (SD = 0.25) s^−1^, and the syllable repetition rate in the series is 9.09 (SD = 0.28) s^−1^.

**Figure 4:**
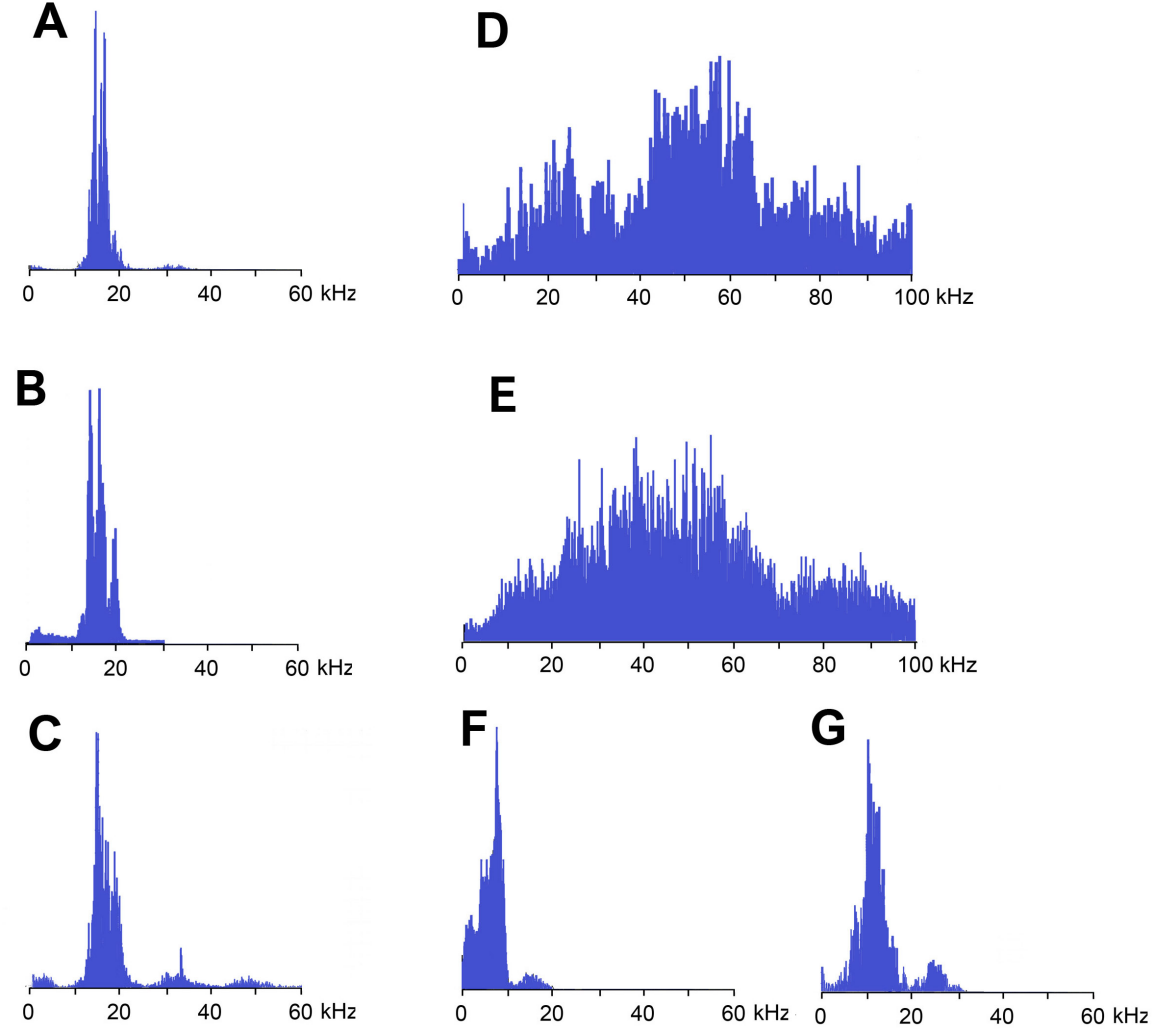
Frequency spectra (in linear scale) of sound signals of *N. nigrispina*. (A) Fast calling song. (B) Slow calling song. (C) Tegminal protest sounds. (D) Male wing protest sounds. (E) Female wing protest sounds; (F) Male mandibular protest sounds. (G) Female mandibular protest sounds. The spectra have the same horizontal scale.

Frequency spectra of calling signals produced in fast and slow rhythms are similar: the main components lie in the range of 10–20 kHz, the dominant frequencies are c. 14–16 kHz (Figs. 4A and 4B).

Intensity of both types of calling songs is c. 110–115 dB SPL at 10 cm from the insect.

In addition to calling sounds, the acoustic repertoire of *N. nigrispina* includes three types of protest signals.

Sounds of the 1st type are made by males with the help of a tegminal sound apparatus in response to mechanical stimuli, for example, to touch. As a rule, these signals are represented by rather long series of identical single syllables (Figs. 3E and 3F; Signal S3). Their duration is c. 40–60 ms and repetition rate is 7.7 (SD = 0.8) s^−1^. However, protest signals occasionally include a series of two or even three syllables.

Frequency spectra (Fig. 4C) cover the range up to 55 kHz, but the dominant frequencies are c. 14 kHz as in spectra of calling songs.

Intensity of these signals is similar to that of calling sounds. In addition to tegminal protest sounds, in response to weak tactile stimuli males and females emit quiet signals (c. 85 dB SPL at 1 cm from the insect) with the help of their hind wings—wing protest sounds (Signals S4 and S5). Occasionally males and females can produce these signals even in the hands of a researcher. In this case, their wings are spread apart and then return to the initial position. These movements are especially noticeable when looking at the insect from above. Tegmina do not rise during sound production, this provides their contact with the wings. Examination of the dorsal surface of abdominal tergites, upper and lower surfaces of hindwings, revealed no distinct stridulatory structures. However, on the lateral sides of abdominal tergites 1 to 3 there are areas covered with small sparse spinules (Figs. 2G and 2H). We carried out experiments in which these spinules were covered with a layer of wax to test whether these structures can be used in stridulation, like the Ander’s organs of *Cyphoderris monstrosa* (Prophalangopsidae) (Woodrow et al., 2021) or abdominal fields with tubercules of representatives of Anostostomatidae (Zhantiev, Kalinkina & Korsunovskaya, 1982) and Gryllacrididae (Field & Bailey, 1997). They showed that the insects in this case were capable to emit wing protest signals. But removal of the tegmina led to major disruption in sound emission: the signal intensity dropped sharply, and only the use of a bat detector helped to establish that several clicks were still produced during movement of hindwings. Obviously, they emit when the lower surface of the hind wings is rubbed against the tergites of either a motionless or a telescopically contracting abdomen.

The SEM images (Figs. 2D and 2F) show that the hind wings are folded in half along the midline and bear several longitudinal veins. No specialized stridulatory structures were found on the abdomen, underside of tegmina (Figs. 2C and 2E), and the upper and the underside surface of hind wings (Figs. 1Q, 2D and 2F)

Sounds of this type in males are irregular syllables of varying duration, amplitude and repetition rate. But in some cases, as in Fig. 3G, insects emit a series of syllables more or less similar in amplitude-temporal structure. In one of the males their duration is 421 (SD = 44) ms, their repetition rate in a series is 1.33 (SD = 0.32) s^−1^. Each male syllable contains several clicks and fragments without amplitude modulation (Fig. 3H). In females, these signals are similar to those in males: these are either irregular syllables, or, if an insect actively reacts to a stimulus, a series of several more or less uniform syllables with a duration of about 400 ms (Figs. 3I and 3J).

Frequency spectra of wing protest sounds in males and females are similar: they are located within both sound and ultrasonic ranges. Dominant frequencies in males are in the band of 45–65 kHz, in females—35–55 kHz (Figs. 4D and 4E).

The third type of protest sounds that we have registered in males (Figs. 3K and 3L), females (Figs. 3M and 3N; Signal S6) and older nymphs during weak tactile stimulation and after it are short clicks which insects produce, apparently using mandibles (Figs. 1M and 1N). We observed these sounds synchronously with movements of their mouthparts. No stridulatory structures have been found on the inner side of labrum. Intensity of these signals is c. 80 dB SPL at 1 cm from insect head. In females these signals consist of one or more clicks. Their duration ranges from several ms to 100 ms (on average 71.2 (SD = 13.9) ms). Females produce them irregularly. Frequency spectra occupy the range of 0–30 kHz (Fig. 4G), dominant frequencies lie near 10 kHz.

In addition, acoustically interacting males from time to time also emit a series of low-amplitude clicks (Figs. 3K and 3L), alternating with series of a calling signal or trills formed by single syllables, similar to tegminal protest signals. Having repeatedly observed these signals (not accompanied by drumming of any body parts on a substrate), we were able to register only one such series with the help of our equipment. The duration of clicks varies from 2 to 17 ms, their repetition rate is 3.4–4 s^−1^. Some of these clicks are double. Dominant frequency in the spectrum lies in the 9–10 kHz region (Fig. 4F) like in the spectrum of female sounds.

A video of a male and female individuals producing protest sounds by use of their hind wings and mouthparts can be seen in Korsunovskaya (2022a) and Korsunovskaya (2022b).

### Vibratory signals

**Figure 5:**
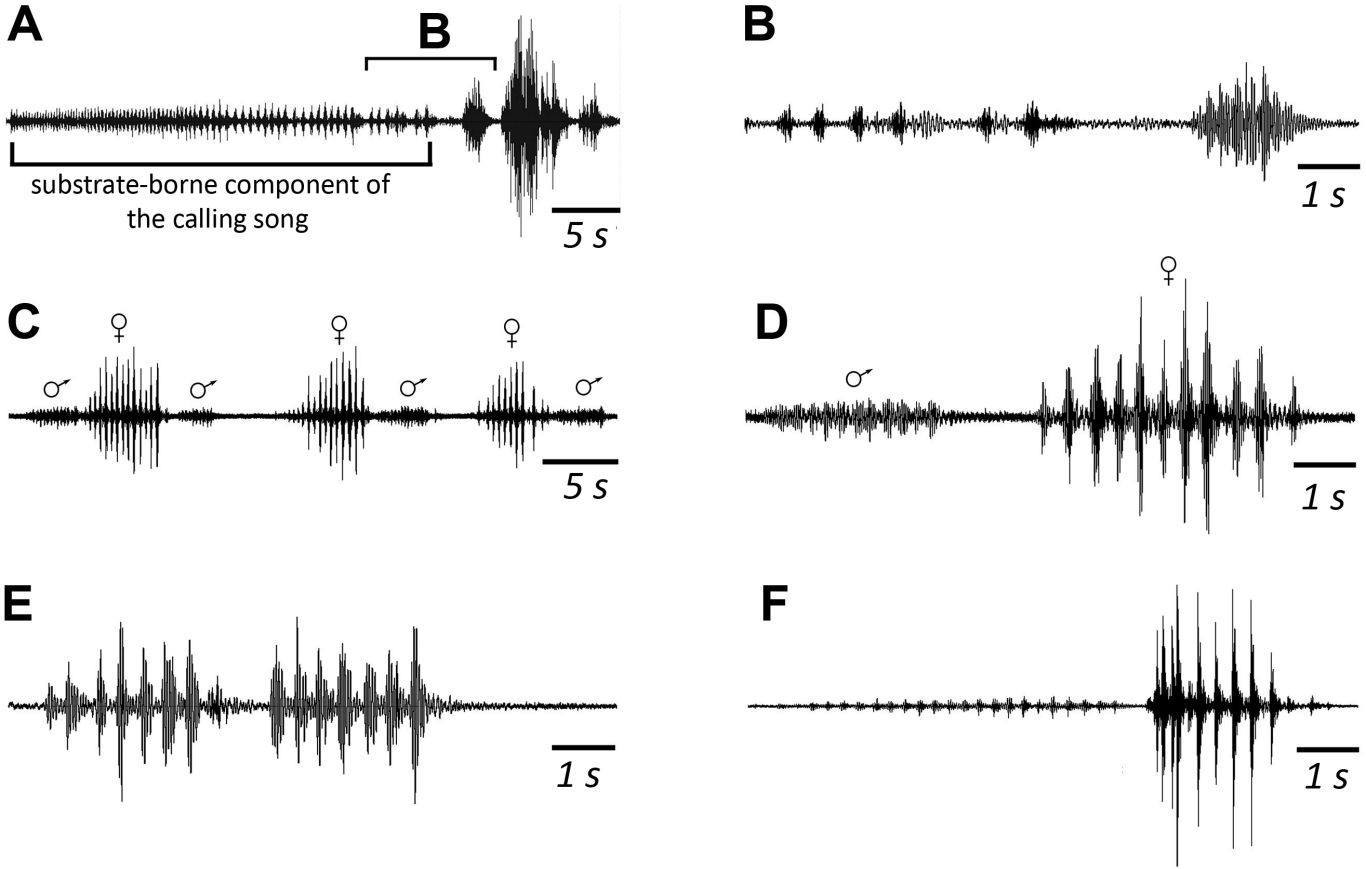
Oscillograms of the vibratory signals of *N. nigrispina*. (A, B) Vibrational component of calling song and final vibratory signal. (C, D) Male and female courtship signals. (E) Male courtship signal. (F) Two postcopulatory male signals. Time scales: 5 s (A, C) and 1 s (B, D–F).

When a pair is formed, up to copulation, the male and female alternately emit a series of 6–10 vibratory signals with a mean duration of 2.9 (male) and 3.7ms (female) respectively. SD = 0.7 ms for both (Figs. 5C and 5D). Repetition rate of their tremulation movements is about 4–5 s^−1^ in males and about 3 s^−1^ in females. The insects produce such signals during tremulation—vibrations of their abdomen in the vertical plane. If range of motion is large, a male can periodically hit a substrate with the tip of its abdomen. A female usually does not touch a substrate (Korsunovskaya, 2022c).

After copulation, a female does not emit signals, but a male continues to produce rhythmic series of vibrations. They last ca. 5–7 s. From time to time, the male produces a series of oscillations of greater amplitude (Fig. 5F), or extremely high-amplitude single blows (with duration of ca. 0.3 s) of its body against a substrate, the insect literally jumps in place during them (Korsunovskaya, 2022d).

## Discussion

Morphological characters of all currently known species of the genus *Nesoecia* are very similar, therefore it is necessary to carry out comprehensive complex studies to confirm their taxonomic status. Investigation of sound and vibratory signals of these species is one of the directions of such research.

Our comparison of the sound signals of the species studied with data on calling sounds of other representatives of *Nesoecia* (Barrientos-Lozano et al., 2020) indicates a very close similarity of their temporal and frequency characteristics. Frequency spectra of calling signals in all five species occupy a narrow range, dominant frequencies are 14–20 kHz. Unfortunately, it is difficult to compare temporal characters of sound signals of these katydids due to their great variability. In our opinion, the most suitable for comparison are such indicators as shape of syllables, pattern of a tooth-impact frequency, as well as syllable repetition rate in the series. Based on these parameters, both fast and slow calling signals of *N. nigrispina* are most similar to those of *N. insolita* and *N. constricta*. The main differences from *N. insolita* signals are in shape of the first syllable, in a slow calling song and in nature of change in frequency of tooth-impacts throughout the first syllable, which may indicate a differences in the structure of pars stridens and/or speed of movement of tegmina during the first syllable in the series in both species. Sounds described by us differ from the signals of *N. constricta* by a greater syllable repetition rate in series: 9.1 (SD = 0.3) s^−1^, while in *N. constricta*, judging by fig. 123 and tab. 1 (Barrientos-Lozano et al., 2020), it is slightly more than 7 s^−1^. There are also differences in the repetition rate of the series: in *N. nigrispina* it is higher and, apparently, overlaps the variability of the signal of *N. constricta*.

The study of vibro-acoustic signaling of *N. nigrispina* has revealed several interesting facts. It turned out that during calling behavior these katydids produce not only sound, but also vibratory signals. However, unlike the neotropical *Nesonotus reticulatus* (Stumpner et al., 2013), they do this not simultaneously, but sequentially, like representatives of several genera of the tribes Cocconotini, Pleminiini (see, for example, Belwood & Morris, 1987; Montealegre-Z & Morris, 1999). Considering that males are capable of emitting a calling signal for several hours, it cannot be concluded that the vibrational component of a calling song was the result of selection pressure from predation. Previously, it has been repeatedly suggested that neotropical katydids reduce their acoustic activity or even completely switch to vibrocommunication under the influence of pressure from predators (bats) (see *e.g.*, Belwood & Morris, 1987; Roemer, Lang & Hartbauer, 2010). Undoubtedly, it is useful for locating a singing male by an approaching female, since communication takes place at night and the possibilities of visual orientation are limited. Experiments on the study of female phonotaxis of bush-crickets *Tettigonia cantans* have confirmed the use of vibratory stimuli for the search a mating partner on vegetation (Latimer & Schatral, 1983).

In addition, it is possible that vibrations after a period of sound calling perform the function of an additional species-specific informative parameter. It can enhance the significance of an acoustic calling song like a factor in prezygotic reproductive isolation. This seems to be quite true, since data on air-borne signaling of sympatric and synchronously singing katydids of the genus *Nesoecia* (Barrientos-Lozano et al., 2020), along with comparison of their songs with calling signals of *N. nigrispina*, indicate a significant similarity in temporal patterns of their songs. Another argument in favor of importance of the vibrational component for recognition of conspecifics is a rather high variability of calling sounds, that was noted not only in *N. nigrispina*, but also in *Xerophyllopteryx fumosa* (Stumpner et al., 2013).

An obligatory component of courtship behavior of *N. nigrispina* are vibratory signals. This type of signal is widespread throughout the acoustically active orthopterans. For example, it is, known among the katydid subfamily Conocephalinae (see, *e.g.*, De Luca & Morris, 1998; Benediktov, 2014; Sarria-S et al., 2016), many Pseudophyllinae (*e.g.*, Morris et al., 1994; Barrientos-Lozano et al., 2020), Phyllophorinae (Korsunovskaya et al., 2020). In *N. nigrispina*, these signals have a clear rhythmic pattern; they are emitted by both males and females. Apparently, besides the function of preparing for copulation and localization of a sexual partner in space, these signals, like vibrations during calling behavior, are capable of performing the function of an additional factor in reproductive isolation. The study of courtship signals in sympatric *Nesoecia* species may confirm or refute this hypothesis. Function of post-copulation vibratory signals appears to result in prevention of premature removal of a spermatophore by a female. Similar, extremely high-amplitude signals were described by us for the giant katydids *Siliquofera grandis* (Korsunovskaya et al., 2020). Of particular interest is the presence of two types of audible calling signals in species of the genus *Nesoecia*: with fast and slow rhythms (Barrientos-Lozano et al., 2020, current article). As our observations have shown, insects produce fast signals during acoustic interaction. However, in some cases, these sounds can be recorded in single males for a rather long time. According to our data, katydids are most likely to emit slow signals when they call spontaneously and isolated. Presence of two types of calling signals in the acoustic repertoire is difficult to explain, since it is still unclear what informative elements the females use to identify conspecific individuals. It can be assumed, by analogy with some phaneropterine bushcrickets (Spooner, 1964; Spooner, 1968; Walker, 2004; Korsunovskaya, 2008; Zhantiev, Korsunovskaya & Benediktov, 2017), that the beginning fast phase of a slow calling signal of *N.nigrispina* or a long period of a fast signal are necessary to identify a conspecific signal, and a slow phase is used during localization of its source and orientation. However, long duration of continuous period of a slow song contradicts this assumption. Obviously, this problem can be solved only by conducting special experiments to study phonotaxis with testing various models of calling signals.

*N. nigrispina* possess wide acoustic repertoire, including several types of signals used in agonistic relationships. We designated them as protest signals. Mechanisms for producing these sounds are different: males with a developed tegminal sound apparatus produce them in response to mechanical stimulation. Sounds of this kind, loud and prolonged, are, as in katydids of the tribe Zichyini (Zhantiev, Korsunovskaya & Byzov, 1995; Elaeva & Korsunovskaya, 2012), apparently aimed at scaring away predators (Masters, 1980; Low, Naranjo & Yack, 2021; Woodrow et al., 2021). Wing protest signals are emitted by both males, and females. Mandibular sounds are produced by males, females and nymphs. These are quiet signals that insects also produce in response to tactile stimuli, but males can make mandibular sounds also in response to any signals from other males. In the latter case, in our opinion, it is possible to talk about agonistic interactions which are aimed at regulation of intrapopulation relationships. The signals from females and nymphs are apparently also intended for conspecific individuals. However, mandibular sounds may also be defensive. Few orthopterans produce defensive sounds by the use of their mandibles. Such signals are described in some species of New Zealand wetas (Anostostomatidae) and the katydids *Mygalopsis marki* (see review Low, Naranjo & Yack, 2021), *Panoploscelis scudderi* and *Gnathoclita vorax* (Hugel, 2019). Ability of females to emit acoustic signals with the help of hind wings was previously known for mecopodine and phyllophorine katydids (Vosseler, 1907; Korsunovskaya et al., 2020). During sound emission, these insects move their hind wings in a vertical plane (*Anoedopoda lamellata*) or flutter them (*Siliquofera grandis*). However, *N. nigrispina* females move hind wings in a horizontal plane. So the list of seven wing (tegminal) non-homologous sound apparatuses of female katydids (Jost & Naskrecki, 2003) can be supplemented.

Thus, acoustic signaling of *N. nigrispina* is the most complex among representatives of Pseudophyllinae studied to date. Reasons for such development of acoustic signaling are the need for protection from predators and, apparently, the need to regulate intrapopulation relationships in various forms of competition. Judging by the absence of specialized sound apparatus for emission of wing and mandibular protest signals, this complication could have arisen in *N. nigrispina* relatively recently in the course of their evolution. We assume that interactions, which lead to an expansion of an acoustic repertoire due to agonistic signals are possible at a sufficiently high population density or at presence of aggregations of conspecifics. However, the latter assumption requires verification in the natural habitat of this species.

## Conclusions

The known species of *Nesoecia* morphologically are very similar; therefore, the results of bioacoustic studies are very important for verification of the species status of congenerics. The study of vibro-acoustic signaling of *N. nigrispina* has shown that these katydids during calling and courtship, produce not only sound, but also vibratory signals. Repertoire of sound signals of *N. nigrispina* is the most extensive among representatives of the family Pseudophyllidae. It includes calling song of two types and three types of protest signals. Males, like other katydids, with a tegminal stridulatory organ, produce sounds of all types. However, the emission of protest signals of the 2nd and 3rd types is carried out with the help of wings and mouth organs (apparently, mandibles). Females can also produce Type 2 and Type 3 protest sounds in the same way as males. No specialized structures for emission of wing and mandibular sounds have been identified, which may indicate that expansion of acoustic repertoire occured in evolution relatively recently. Perhaps this has arosen owing the need of both performing a protective function against attacks of predators, and regulating agonistic intrapopulation relations. Vibratory signals are emitted by individuals of both sexes during courtship, males, completing their calling signal cycle and after copulation. It is possible to propose that vibratory signals are an additional factor of reproductive isolation in sympatric species, since calling sound signals in representatives of the genus *Nesoecia* are similar and exhibit significant variability. Type and parameters of a calling signal the females use to identify their conspecific sexual partners should be revealed by special studies.

## Supplemental Information

10.7717/peerj.13749/supp-1 Signal S1 Signal S1 male fast calling song *N. nigrispina* male fast calling song, sampling rate 200 kHz

10.7717/peerj.13749/supp-2 Signal S2 Signal S2 male slow calling song *N. nigrispina* male slow calling song, sampling rate 200kHz

10.7717/peerj.13749/supp-3 Signal S3 Signal S3 male tegminal protest sounds * N. nigrispina* male tegminal protest sounds, sampling rate 96 kHz

10.7717/peerj.13749/supp-4 Signal S4 Signal S4 male wing protest sounds * N. nigrispina* male wing protest sounds, sampling rate 96kHz

10.7717/peerj.13749/supp-5 Signal S5 Signal S5 female wing protest sounds * N. nigrispina* female wing protest sounds, sampling rate 200kHz

10.7717/peerj.13749/supp-6 Signal S6 Signal S6 female mandibular protest sounds * N. nigrispina* female mandibular protest sounds, sampling rate 96kHz

## Additional Information and Declarations

### Data Availability

The following information was supplied regarding data availability: The data is available at Zenodo: Korsunovskaya Olga. (2022, January 28). Video of male wing and mandibular protest sounds of the katydid, *Nesoecia nigrispina* (Orthoptera, Pseudophyllinae). Zenodo. https://doi.org/10.5281/zenodo.5913615. Korsunovskaya Olga. (2022, January 28). Video of precopulatory tremulation during courtship in *Nesoecia nigrispina* (Orthoptera, Pseudophyllinae). Zenodo. https://doi.org/10.5281/zenodo.5913721. Korsunovskaya Olga. (2022, January 28). Video of male postcopulatory tremulation of the katydid, *Nesoecia nigrispina* (Orthoptera, Pseudophyllinae). Zenodo. https://doi.org/10.5281/zenodo.5913738. Korsunovskaya Olga. (2022, January 28). Video of female wing and mandibular protest sounds of the katydid, *Nesoecia nigrispina* (Orthoptera, Pseudophyllinae). Zenodo. https://doi.org/10.5281/zenodo.5913684.
